# Automatic Evaluation of Speech Rhythm Instability and Acceleration in Dysarthrias Associated with Basal Ganglia Dysfunction

**DOI:** 10.3389/fbioe.2015.00104

**Published:** 2015-07-24

**Authors:** Jan Rusz, Jan Hlavnička, Roman Čmejla, Evžen Růžička

**Affiliations:** ^1^Department of Circuit Theory, Faculty of Electrical Engineering, Czech Technical University in Prague, Prague, Czech Republic; ^2^Department of Neurology and Centre of Clinical Neuroscience, First Faculty of Medicine, Charles University in Prague, Prague, Czech Republic

**Keywords:** Parkinson’s disease, Huntington’s disease, atypical parkinsonian syndromes, dysarthria, speech and voice disorders, rhythm, oral festination, acoustic analyses

## Abstract

Speech rhythm abnormalities are commonly present in patients with different neurodegenerative disorders. These alterations are hypothesized to be a consequence of disruption to the basal ganglia circuitry involving dysfunction of motor planning, programing, and execution, which can be detected by a syllable repetition paradigm. Therefore, the aim of the present study was to design a robust signal processing technique that allows the automatic detection of spectrally distinctive nuclei of syllable vocalizations and to determine speech features that represent rhythm instability (RI) and rhythm acceleration (RA). A further aim was to elucidate specific patterns of dysrhythmia across various neurodegenerative disorders that share disruption of basal ganglia function. Speech samples based on repetition of the syllable /pa/ at a self-determined steady pace were acquired from 109 subjects, including 22 with Parkinson’s disease (PD), 11 progressive supranuclear palsy (PSP), 9 multiple system atrophy (MSA), 24 ephedrone-induced parkinsonism (EP), 20 Huntington’s disease (HD), and 23 healthy controls. Subsequently, an algorithm for the automatic detection of syllables as well as features representing RI and RA were designed. The proposed detection algorithm was able to correctly identify syllables and remove erroneous detections due to excessive inspiration and non-speech sounds with a very high accuracy of 99.6%. Instability of vocal pace performance was observed in PSP, MSA, EP, and HD groups. Significantly increased pace acceleration was observed only in the PD group. Although not significant, a tendency for pace acceleration was observed also in the PSP and MSA groups. Our findings underline the crucial role of the basal ganglia in the execution and maintenance of automatic speech motor sequences. We envisage the current approach to become the first step toward the development of acoustic technologies allowing automated assessment of rhythm in dysarthrias.

## Introduction

Speech represents the most complex acquired motor skill requiring the precise coordination of more than 100 muscles (Duffy, 2013). Speech is thus an important indicator of motor function and movement coordination, and can be extremely sensitive to neurological disease. In particular, speech may be affected due to disturbances in the basal ganglia. It is widely recognized that the basal ganglia are involved in planning, programing, and execution of motor tasks. It has been hypothesized that they also play an important role in the control of speech, including the selection of motor programs, execution, and sensory feedback (Ho et al., 1999; Kent et al., 2000; Graber et al., 2002).

Parkinson’s disease (PD) is a common neurological disorder that is associated with dysfunction of the basal ganglia and arises due to the degeneration of dopaminergic neurons, leading to the principal motor manifestations of bradykinesia, rigidity, and resting tremor (Hornykiewicz, 1998). Atypical parkinsonian syndromes (APS), such as progressive supranuclear palsy (PSP) and multiple system atrophy (MSA), represent the most common forms of neurodegenerative parkinsonism after PD (Schrag et al., 1999). PSP and MSA differ from PD by more widespread neuronal atrophy, atypical clinical signs, more rapid disease progression, and poor response to dopamine replacement therapy. In addition, in ephedrone-induced parkinsonism (EP), manganese intoxication leads to a rapidly progressive, irreversible, and levodopa-resistant parkinsonian syndrome with features of dystonia (Levin, 2005; Selikhova et al., 2008). Although Huntington’s disease (HD) also primarily affects the basal ganglia, differing pathophysiology results in involuntary movements termed chorea, as well as psychiatric disturbances and cognitive deficits resulting in dementia (Roos, 2010). Altogether, these five neurological disorders represent a variety of motor and non-motor deficits associated with impaired function of the basal ganglia.

There is growing evidence that PD is associated with abnormalities in the performance of simple, automated, repetitive movements, such as finger tapping, diadochokinesia, and gait (O’Boyle et al., 1996; Takakusaki et al., 2008). These deficits have been suggested to be induced by impaired planning, preparation, and execution of motor sequences, particularly as a consequence of basal ganglia impairment (Iansek et al., 1995). Previous studies have revealed that patients with impaired function of the basal ganglia showed similar instabilities in speech production. In particular, PD, HD, and PSP manifest difficulties in the steady performance of single syllable repetition without speed alterations (Skodda et al., 2010, 2012, 2014), likely due to shared pathophysiology with similar dysfunctional neural circuits. Nevertheless, pace stability in MSA and EP remain unknown.

Among the various rhythm irregularities, PD patients demonstrate a tendency for pace acceleration during both simple and more complex utterances (Skodda and Schlegel, 2008; Skodda et al., 2010). Pace acceleration, also known as oral festination, is a frequent component of axial impairment in PD and is thought to share similar pathogenic mechanisms with gait festination (involuntary acceleration and progressive step shortening; Moreau et al., 2007). This hypothesis is supported by correlation reported between several aspects of speech and gait disturbances in PD (Moreau et al., 2007; Cantiniaux et al., 2010; Skodda et al., 2011a). However, evidence related to speech acceleration is primarily based on observations in PD (Skodda and Schlegel, 2008; Skodda et al., 2010), while a targeted investigation of oral festination in APS or in HD has not been performed. Therefore, the evaluation of speech rhythm acceleration (RA) in PD, APS, and HD may provide additional insight into the pathophysiology of basal ganglia dysfunction.

There is thus a need for reliable, cost-effective, and automatic methods allowing the precise and objective assessment of various speech patterns, such as rhythm abnormalities. Increasing computational power has enabled a higher level of automation in speech assessment. Indeed, a number of studies have introduced novel methods for automatic acoustic speech analyses in various neurological disorders. Most effort has been put into the automatic investigation of dysphonic features of dysarthria in PD through the sustained phonation task (Little et al., 2009; Tsanas et al., 2012). Additional research has demonstrated that articulatory disorders in PD can be reliably assessed through a rapid syllable repetition paradigm (Novotny et al., 2014). Interestingly, acoustic speech analyses can be used as a promising instrument in the differential diagnosis of various forms of parkinsonism (Rusz et al., 2015). Moreover, longitudinal objective monitoring of speech appears to be a more sensitive marker of disease progression than available clinical scales in speakers with cerebellar ataxia (Rosen et al., 2012).

Current methods enabling the objective evaluation of rhythm in dysarthrias are semi-automatic and require hand-labeling, or at a minimum, user control of the analysis procedure. One approach to objectively assess rhythm in dysarthrias is based upon various measurements of vocalic and consonantal intervals that are extracted from connected speech, particularly short phrases or sentences, where the boundaries between vowels and consonants need to be identified and hand-labeled by visual inspection of speech waveforms and spectrograms (Liss et al., 2009). Another approach is based on metrics derived using the intervals obtained from a syllable repetition paradigm, where the intervals between two syllables are identified and hand-labeled using the oscillographic sound pressure signal as the periods from onset of one vocalization until the following vocalization (Skodda et al., 2010). Such hand-labeling is considerably time consuming and requires an experienced investigator. However, to the best of our knowledge, there is currently no robust algorithm for the automatic evaluation of speech rhythm in dysarthric speakers.

Therefore, the aim of the present study was to develop a robust signal processing technique allowing the automatic detection of spectrally distinctive nuclei of syllable vocalizations and to design acoustic features representing rhythm instability (RI) and RA. The subsequent aim of our study was to elucidate specific patterns of dysrhythmia across various neurodegenerative disorders with functional disruption of the basal ganglia.

## Materials and Methods

### Subjects

The participants in the present study were originally recruited for previous studies (Rusz et al., 2014a,b, 2015), however, the method of automatic rhythm evaluation as well as rhythm characteristics were not reported. Data were obtained from a total of 109 subjects, 22 of which were diagnosed with PD (10 men, 12 women), 11 with PSP (9 men, 2 women), 9 with MSA (3 men, 6 women), 24 with EP (24 men), and 20 with HD (9 men, 11 women). Additionally, 23 subjects (12 men, 11 women) with no history of neurological or communication disorders participated as healthy controls (HC). The diagnosis of PD was established by the UK Parkinson’s Disease Society Bank Criteria (Hughes et al., 1992), PSP by the NINDS-PSP clinical diagnosis criteria (Litvan et al., 1996), MSA by the consensus diagnostic criteria for MSA (Gilman et al., 2008), EP by the history of ephedrone use and typical clinical and magnetic resonance findings (Rusz et al., 2014a), and HD by clinical and genetic testing (Huntington Study Group, 1996). PD subjects were on stable medication for at least 4 weeks before the testing and were investigated in the on-medication state. PSP and MSA patients received various doses of levodopa alone or in combination with different dopamine agonists and/or amantadine. The majority of EP patients were free of any neurological therapy. Most HD patients were treated by benzodiazepines, antipsychotics, amantadine, and antidepressants, in monotherapy or in various combinations. In order to ensure that the results were not influenced by severe respiratory problems, the inclusion criteria were determined as the ability to sustain prolonged phonation for at least 6 s and to perform at least 20 syllables in sequence.

Disease duration was estimated based on the self-reported occurrence of first motor symptoms. Severity of motor involvement in PD patients was scored by the Unified Parkinson’s Disease Rating Scale motor subscore (UPDRS III; Stebbing and Goetz, 1998). APS patients were rated by the natural history and neuroprotection in Parkinson plus syndromes–Parkinson plus scale (NNIPPS; Payan et al., 2011). HD patients were assessed using the motor score of the Unified Huntington’s Disease Rating Scale (UHDRS; Huntington Study Group, 1996). Thus, the perceptual severity of speech impairment was established using speech/dysarthria items of appropriate clinical scales. Each participant provided written, informed consent, and the study was approved by the Ethics Committee of the General University Hospital in Prague, Czech Republic. Participant characteristics are summarized in Table 1.

**Table 1:** **Clinical characteristics of participants**.

Group (dominant type of dysarthria)	Age (years)	Disease duration (years)	Disease severity ^a^	Speech severity ^b^
	Mean/SD (range)	Mean/SD (range)	Mean/SD (range)	Mean/SD (range)
PD (hypokinetic)	64.4/9.6 (48–82)	9.3/5.5 (1–24)	15.9/7.6 (6–34)	0.7/0.7 (0–2)
PSP (hypokinetic-spastic)	65.3/5.2 (54–71)	3.5/1.3 (1–5)	66.2/29.0 (19–116)	1.9/0.9 (0–3)
MSA (ataxic-hypokinetic)	59.6/3.6 (55–65)	3.8/1.5 (2–6)	86.6/17.6 (63–123)	1.9/0.6 (1–3)
EP (hyperkinetic-hypokinetic)	39.7/5.0 (28–48)	3.8/2.0 (1–11)	85.6/32.6 (42–166)	1.7/1.0 (0–4)
HD (hyperkinetic)	49.6/14.8 (23–67)	6.4/3.0 (2–13)	23.6/11.8 (3–42)	0.6/0.5 (0–1)
HC (none)	64.4/10.5 (41–81)	–	–	–

*^a^Scores on the Unified Parkinson’s Disease Rating Scale III (UPDRS III) for PD (ranging from 0 to 108), natural history and neuroprotection on Parkinson (NNIPPS) for APS (ranging from 0 to 332), and Unified Huntington’s Disease Rating Scale (UHDRS) motor subscore (ranging from 0 to 124), higher scores indicate more severe disability*.

*^b^Scores on the UPDRS III 18 speech item for PD, NNIPPS Bulbar–pseudobulbar signs subscale (item 3) for APS, and UHDRS dysarthria item*.

*All scores represent speech motor examination and range from 0 to 4, where 0 represents normal speech, 1 mildly affected speech, 2 moderately impaired speech (still intelligible), 3 markedly impaired speech (difficult to understand), and 4 unintelligible speech*.

### Speech recordings

Speech recordings were performed in a quiet room with a low ambient noise level using a head-mounted condenser microphone (Bayerdynamic Opus 55, Heilbronn, Germany) situated approximately 5 cm from the mouth of each subject. Speech signals were sampled at 48 kHz with 16-bit resolution. Each participant was instructed to repeat the syllable /pa/ at least 20 times at a comfortable, self-determined, steady pace without acceleration or deceleration. All subjects performed the syllable-repetition task twice. The syllable /pa/ was chosen with respect to previous research (Skodda et al., 2010), and was preferred for several reasons. The unvoiced consonant with short voice onset time and minimal energy is represented by /p/, which ensures stop closure and therefore allows robust detection even in speakers with faster tempo of repetitions. The syllable /pa/ also requires minimal tongue movement and thus is a suitable task for patients with more severe dysarthria, where the use of more articulatory-demanding consonants could influence rhythm performance.

### Automatic algorithm for detection of syllables

**Figure 1:**
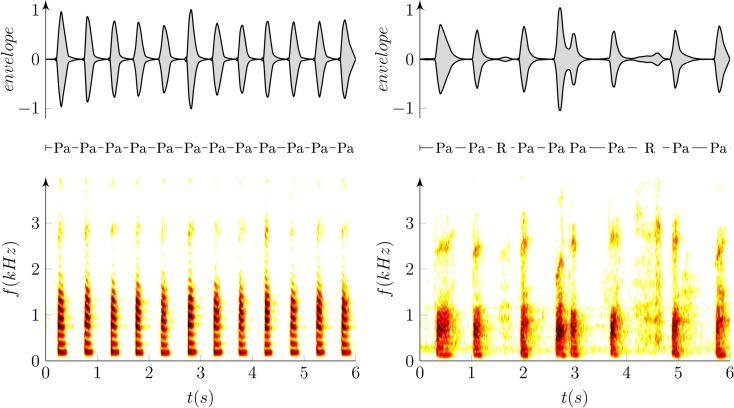
**Example of oscillographic sound pressure signals (up) and their respective spectrograms (down) of the repetition of syllable /pa/ in a healthy (left; RA = −0.6 ms/s, RI = 4%) and dysarthric speaker (right; RA = 73.6 ms/s, RI = 24%)**. “Pa” represents the syllable /pa/ whereas “R” depicts excessive inspirations due to respiratory problems, and arrows show detected time labels.

**Figure 2:**
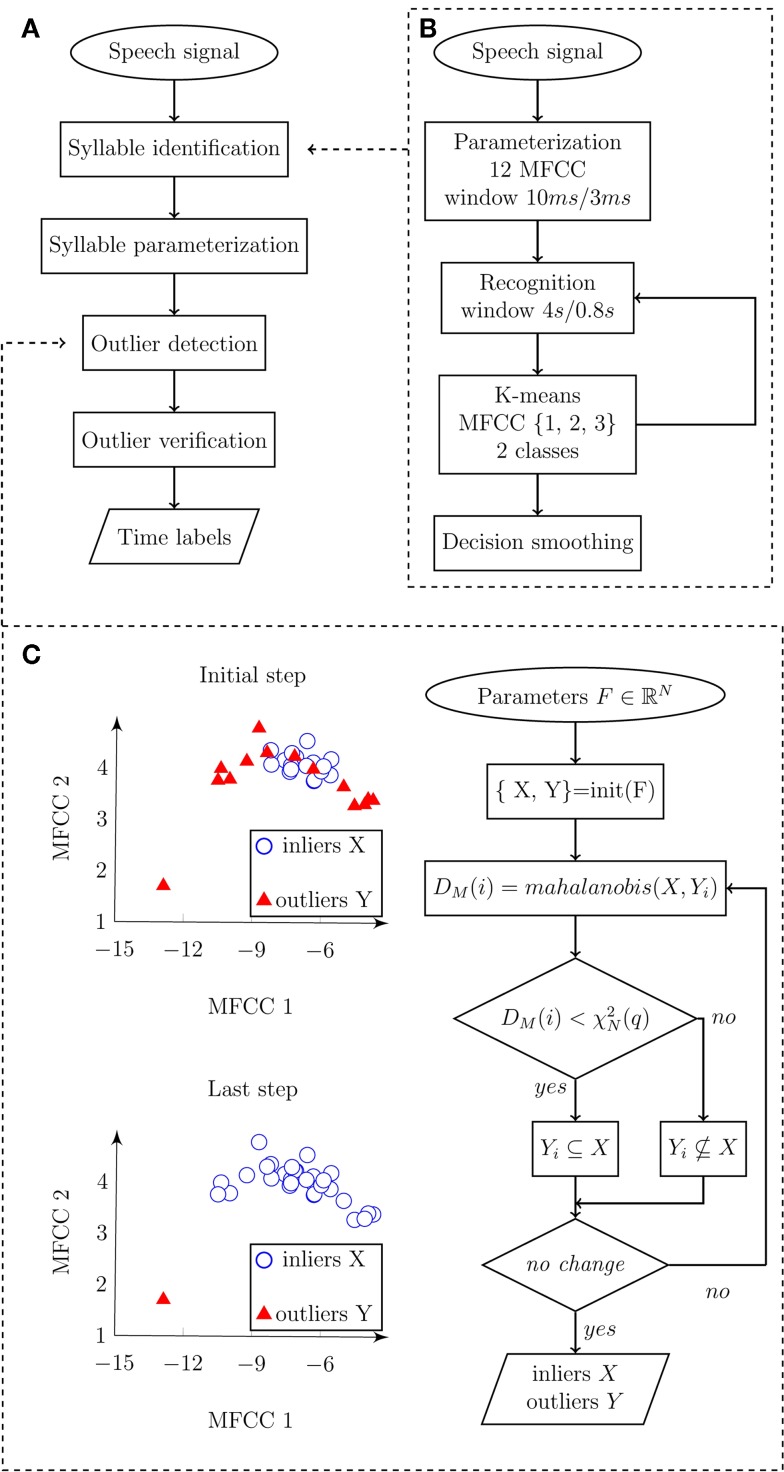
**Flowchart diagram depicting an automatic algorithm for syllable detection**. **(A)** Main diagram describing the principle steps of syllables detection. Input represents speech signal, and output represents time labels related to individual syllable vocalizations. **(B)** Detail of syllable identification procedure. Input speech signal is parameterized and subsequently classified into speech/pause classes. Output of the algorithm represents detected syllable boundaries. **(C)** Details of outlier detection. Input is represented by syllables parameterized in three-dimensional space *F* using the first three MFCC, where *X* represents inliers (detected syllables), *Y* represents outliers (non-speech sounds), *D*_M_(*i*) is Mahanalobis distance of observation *i*, $\chi_{N}^{2}\left(q\right)$ is the value of two degrees of freedom in quantile *q*. Output consists of marked inliers and outliers.

**Figure 3:**
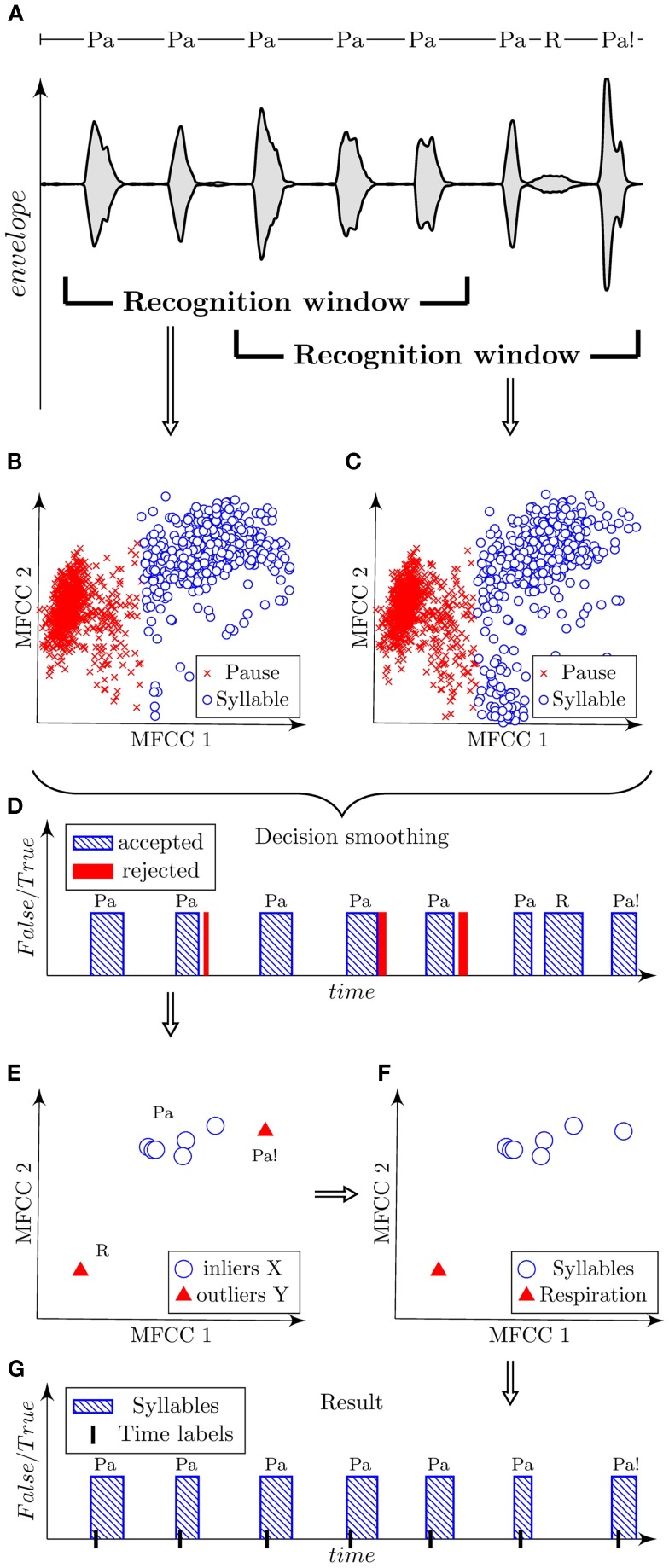
**Illustration of automatic algorithm for the detection of syllables using a sample of dysarthric speech containing syllables “Pa,” respirations “R,” and dissimilarly articulated syllables “Pa!.”** Signal is shown using the oscillographic sound pressure envelope with marked positions of the recognition window **(A)**. Classified parametric space of the first three MFCC is plotted in scatter diagrams for each corresponding position of the recognition window **(B,C)**. Decision smoothing of classified signal is plotted in graph with marking of rejected detections **(D)**. Parameterized syllables and highlighted detected outliers are shown in scatter diagram **(E)**. Verified outliers are illustrated in scatted diagram **(F)**. Final result is shown in graph with marked time labels related to highest energy peak of each syllable **(G)**.

Dysarthric speech typically manifests unstable loudness of voice, imprecise syllable separation, and higher noise levels in occlusions and respirations, making the detection of syllables in the rhythm test difficult. However, the precise identification of syllables requires detection sensitive to imprecisely articulated syllables but insensitive to voiced or noised gaps and inspirations between syllables at the same time (Figure 1). The proposed method overcomes these contradictions in two steps. The first step consists of sensitive syllable detection based on adaptive recognition. The second step determines and removes error detections that are mainly caused by respirations (mostly audible inspirations) and non-speech sounds (mostly turbulent airflow of incomplete occlusion and tongue clicks). Respirations differed from non-speech sounds by prolongation between syllables and a distinctive spectral envelope with formant frequencies above 1 kHz and durations typically longer than 100 ms. Figure 2A shows the main principle of the algorithm whereas Figure 3 highlights the overall decision process overlaid on acoustic input.

#### Syllable Identification

Frequencies higher than 5 kHz are redundant in the precise detection of syllable nuclei; therefore, we decimated the signal into a sampling frequency of 10 kHz. The signal was parameterized to 12 Mel-frequency cepstral coefficients (MFCC) inside a sliding window of 10 ms length, 3 ms step, and hamming weighting. Subsequently, we searched for a low frequency spectral envelope, which can be described using the first three MFCC. Short adaptation time is desirable as high sensitivity is required. Short adaptation was provided by the recognition window. Therefore, syllables were classified using the first three MFCC inside a recognition window of 4 s length and 800 ms step. The length of 4 s was determined experimentally and ensures that at least one syllable will be included in the recognition window. The window length of 4 s with 800 ms step size is optional and can be changed if necessary. In general, a shorter window provides greater sensitivity but results in more false detections. A bimodal multidimensional normal distribution of the first three MFCC inside the recognition window was assumed. The presumption for classification is that syllables and pauses should have the same variance, and therefore we preferred *k*-means rather than the EM algorithm, as the EM algorithm tends to converge into local optima. The component with higher mean of the first MFCC (related to power) represents syllables. The decision was smoothed using a median filter of the fifth order. Pulses shorter than 30 ms and pauses shorter than 80 ms were rejected. Figure 2B highlights the syllable identification process using a flow diagram.

#### Syllable Parameterization

As the characteristics of the signal were unknown and we expected false-positive (FP) detections, each syllable was parameterized to the vector of means of each of the first three MFCC and each syllable observation was judged in relation to others.

#### Outlier Detection

The purpose of this step was to recognize true syllables (inliers *X*) and false detections (outliers *Y*) from previously identified syllables. The presumption was that observations of syllables *X* will form a normal distribution in the space *F* of the first three MFCC. FP detections represented mostly by audible inspirations have a different spectral envelope and should act as outliers to this distribution. The distance relative to the variance and the mean of normal distribution *X* was measured using Mahalanobis distance:

$$D_{\text{M}}\left(x\right)=\sqrt{{\left(x-\mu \right)}^{\text{T}}\cdot S^{-1}\cdot \left(x-\mu \right)},$$

where *D*_M_*(x)* is Mahalanobis distance between the observation *x* and the distribution *X*, μ is mean of the distribution *X*, and *S* is the covariance matrix of the distribution *X*. A normal distribution will form the χ^2^ distribution of Mahalanobis distances with *N* degrees of freedom, where *N* represents the number of dimensions. It is common to presuppose outliers in quantile of approximately *q* = 0.975 and get an optimal threshold as $\chi_{\text{N}}^{2}\left(q\right)$. However, our case consists of a very small number of observations and outliers were therefore identified using three steps. In the initiation step, mutual Mahalanobis distances of all detections were measured. Inliers *X* were frequently identified under the low quantile *q* = 0.3. Outliers *Y* occurred above this quantile. In the identification step, Mahalanobis distances between each observation of *Y* and distribution of *X* were measured. Outliers were identified above the empirical quantile, *q* = 0.5. In the last repetition step, the identification step was iteratively repeated until no new outliers were identified or a maximal number of iterations were counted. The algorithm converges on a very precise identification of outliers in a chosen quantile. The outlier verification process is depicted in Figure 2C.

#### Outlier Verification

Diversely articulated syllables (too quiet or too loud) may exhibit a different spectral envelope and may be detected as outliers in our very low quantile. Therefore, the outlier was verified in terms of power. The speech signal was filtered using a Chebyshev’s filter of the fifth order in 100–500 Hz band pass. The power of the filtered signal was calculated in a sliding window of 10 ms length, 3 ms overlap, and hamming weighting. Each syllable *P*_X_ was parameterized to the mean of power and each outlier *P*_Y_ to the maximum value of the power. Subsequently, the outlier *Y(i)* was rejected on 95% population level of one-sided Chebyshev’s inequality. In other words, if the outlier *Y(i)* belonged to the energy range of 95% of inliers *X*, it was reclassified to *X* meeting the condition:

$$P_{\text{Y(i)}}>E\left(P_{\text{X}}\right)-4\sigma \left(P_{\text{X}}\right),$$

where *E* denotes mean and σ is the SD.

#### Time Labels

Syllables were described into labels as time of highest filtered energy peak of each syllable.

### Reference hand labels

To obtain feedback for the evaluation of reliability of the proposed automatic algorithm, manual syllable annotations of all available utterances were performed blindly, i.e., without labels obtained by the automatic algorithm. Manual labels were performed after algorithm was designed and were not used for tuning of the algorithm in order to maximize agreement with the hand-labeled measures. In each syllable vocalization, the positions of two events including the initial burst of the consonant /pa/ and occlusion of the vowel /a/ were annotated. This approach was preferred as it is difficult to hand-label the correct position of maximal energy during each syllable by visual inspection of speech waveforms. Previously designed rules were used as a foundation for our labeling criteria (Novotny et al., 2014). The time domain was preferred for the specification of burst onset. In the case of multiple bursts, the initial burst was marked. The frequency domain was used for the identification of vowel occlusion, where the energy of fundamental as well as the first three formant frequencies slowly weakens. The second formant vowel offset was considered as the best indicator of occlusion onset.

To determine final time labels using hand-label annotations, especially for the calculation of rhythm metrics, time of power maxima between burst onset and vowel occlusion was calculated for each syllable vocalization. Such hand time labels ensure a certain similarity to labels obtained using the automatic algorithm.

For a detailed analysis of the classification accuracy of the proposed algorithm, manual annotation of all respirations and non-speech sounds across all available utterances was also performed. The respirations and non-speech sounds were identified mainly using the frequency domain and audio perception.

### Rhythm features

The pace rate (PR) was calculated as a number of syllable vocalizations per second. Based on the time labels, we implemented four measurements to evaluate RI and RA. The measure of rhythm pace stability was defined using the coefficient of variation (COV_5–20_), which was calculated for intervals 5–20 in relation to the average interval length of the first four utterances (avIntDur_1–4_) using the formula $\text{CO}{\text{V}}_{5-20}=s_{\text{5}-\text{2}0}∕\left[\left(\text{avIntDu}{\text{r}}_{\text{1}-\text{4}}\right)∕\sqrt{16}\right]\times 100$, where σ is the SD (Skodda et al., 2010). In addition, the measure of pace acceleration was defined as the difference between average interval lengths of the intervals 5–12 (avIntDur_5–12_) and 13–20 (avIntDur_13–20_), normalized by the average reference interval length using the formula PA = 100 × (avIntDur_5–12_ − avIntDur_13–20_)/avIntDur_1–4_, with values >1 indicating acceleration of rhythm (Skodda et al., 2010). The predisposition of PA is that avIntDur_13–20_ will be considerably shorter than avIntDur_5–12_ with accelerated speech performance.

**Figure 4:**
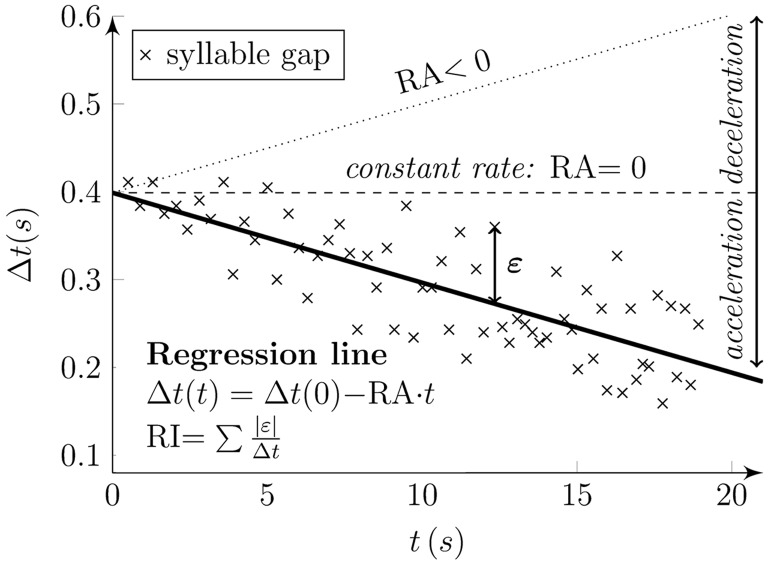
**Principles of designed rhythm features**. Syllable gaps (“*x*” marks) were determined as the duration between two subsequent syllables (Δ*t*) at the time of their occurrence (*t*). The regression line of syllable gaps (solid line) describes the rhythm features. The gradient of the line (RA) denotes rhythm acceleration. The RA results in negative values for rhythm acceleration (solid line), positive values for rhythm deceleration (dotted line), and zero values for constant rhythm (dashed line). Rhythm instability (RI) is obtained as the sum of absolute deviations of each observation in term of gasp duration (Δ*t*) from the regression line (epsilon) and is weighted to the total speech time.

Furthermore, we proposed two alternative features to evaluate RI and RA with a similar function as proposed previously (Skodda et al., 2010). We determined syllable gaps as the duration between two consecutive syllables. RI was calculated as the sum of absolute deviations of each observation in terms of gasp duration from the regression line, weighted to the total speech time. RA was then defined as the gradient of the regression line obtained through regression performed on these syllable gaps, with values >0 indicating accelerated rhythm performance. Figure 4 illustrates the principles of the designed acoustic rhythm features.

### Statistics

To estimate the reliability of the proposed automatic algorithm, each label obtained by the automatic algorithm was compared if it fits into the appropriate time interval between consonant burst and vowel occlusion, as determined using manual annotation. An automatic label that did not fit into an appropriate syllabic time interval was counted as an error. A syllabic time interval with no automatic label was counted as an error. Only one automatic label could be associated with one appropriate syllabic time interval, other automatic labels in the same interval were counted as errors. The overall percentage accuracy (ACC) of the algorithm for each utterance was calculated as:

$$\text{ACC}=100-100\times \frac{\text{number of error detections by algorithm}}{\text{number of syllables determined using manual annotation}}.$$

In addition, the FP percentage score was counted as:

$$\text{FP}=100\times \frac{\text{number of erroneously detected respirations and nonspeech sounds by algorithm}}{\text{number of syllables determined using manual annotation}}.$$

The false negative (FN) percentage score was obtained as:

$$\text{FN}=100\times \frac{\text{number of unidentified syllables by algorithm}}{\text{number of syllables determined using manual annotation}}.$$

Final values of rhythm features used for statistical analyses were calculated by averaging the data for each participant obtained in two vocal task runs. To assess group differences, each acoustic metric was compared across all six groups (PD, PSP, MSA, EP, HD, HC) using a Kruskal–Wallis test with *post hoc* Bonferroni adjustment. The Spearman correlation was applied to find relationships between variables. With respect to the explorative nature of the current study, adjustment for multiple comparisons with regard to correlations was not performed and the level of significance was set to *p* < 0.05.

## Results

Table 2 shows the occurrence of respirations and non-speech sounds as well as the overall classification accuracy of the designed algorithm across all investigated groups. The overall classification accuracy of the proposed algorithm was found to be very high, with a score of 99.6 ± 2.0%. FP error consisted of 85% of respirations and 15% of non-speech sounds. The greatest occurrence of respirations was observed in the HD group. Non-speech sounds were most frequent in the APS and HD groups. Correlations between rhythm features based on automatic time labels and manual reference time labels showed very high reliability (*r* = 0.95–0.99, *p* < 0.001).

**Table 2:** **Occurrence of respiration, non-speech sounds and classification accuracy of the proposed algorithm across individual groups**.

	HC	PN	EP	HD	MSA	PSP	All
**Occurrence of respirations**							
Mean/SD (%) (range)	2.50/4.27 (0–20)	3.06/3.99 (0–12)	3.92/5.50 (0–24)	6.20/6.57 (0–25)	4.18/6.69 (0–24)	3.07/4.40 (0–13)	3.77/5.34 (0–25)
**Occurrence of non-speech sounds**							
Mean/SD (%) (range)	4.90/3.98 (0–13)	5.64/4.59 (0–17)	10.90/11.57 (0–45)	8.60/7.88 (0–36)	7.21/7.31 (0–22)	6.62/4.09 (0–13)	7.43/7.78 (0–45)
**Algorithm accuracy**							
False positives FP^a^
Mean/SD (%) (range)	0/0 (0–0)	0/0 (0–0)	0.39/1.53 (0–19)	0.49/2.15 (0–12)	0/0 (0–0)	0/0 (0–0)	0.18/1.18 (0–19)
False negatives FN^b^
Mean/SD (%) (range)	0/0 (0–0)	0.11/0.72 (0–5)	0.68/2.75 (0–14)	0.11/0.66 (0–4)	0/0 (0–0)	0.69/2.00 (0–9)	0.27/1.55 (0–14)
Overall ACC
Mean/SD (%) (range)	100/0 (100–100)	99.89/0.72 (95–100)	98.92/3.06 (86–100)	99.40/2.22 (88–100)	100/0 (100–100)	99.30/2.00 (92–100)	99.56/1.93 (86–100)

*^a^False positives represent percentage of respirations and non-speech sounds identified by algorithm as syllables*.

*^b^False negatives represent percentage of syllables that were not identified by algorithm*.

**Figure 5:**
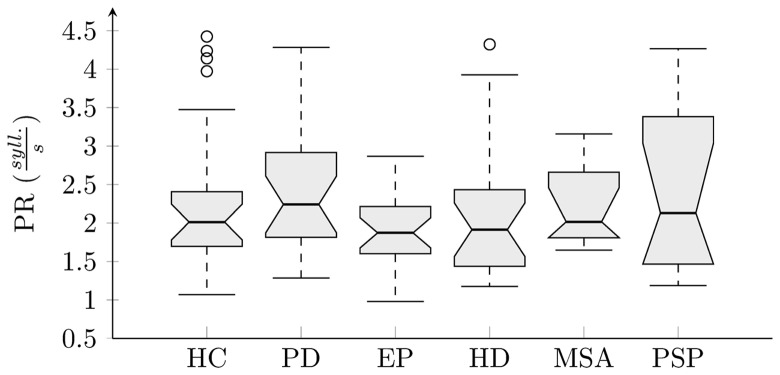
**Boxplot of pace rate analysis across individual groups**.

**Figure 6:**
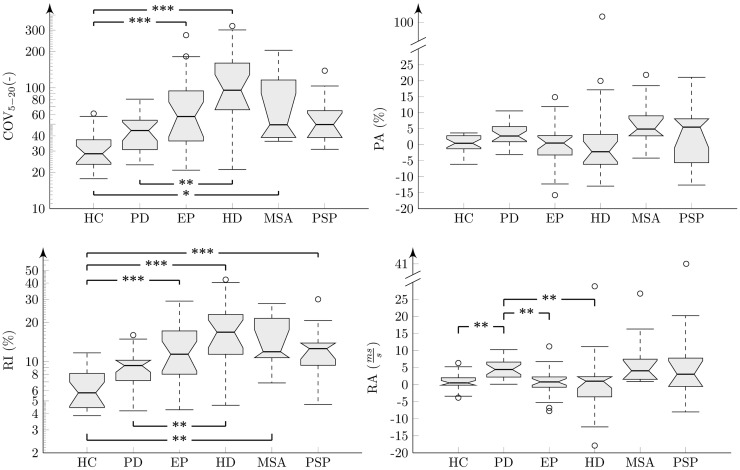
**Results of acoustic rhythm analyses across individual groups shown in boxplots**. Comparison between groups after *post hoc* Bonferroni adjustment: *p < 0.05; **p < 0.01; ***p < 0.001. The “*y*” axis for COV and RI features are in the logarithmic scale.

The results of analyses across all groups and each investigated feature were interpreted using boxplots (Figures 5 and 6). There were no statistically significant differences for PR across investigated groups ($\chi_{5,108}^{2}=5.1$, p = 0. 40, η^2^ = 0. 05) (Figure 5). Statistically significant differences between groups were found for all rhythm metrics including COV_5–20_ ($\chi_{5,108}^{2}=40.3$, p < 0. 001, η^2^ = 0. 37), PA $\chi_{5,108}^{2}=13.1$, p = 0. 02, η^2^ = 0. 12), RI ($\chi_{5,108}^{2}=44.2$, p < 0. 001, η^2^ = 0. 41), and RA ($\chi_{5,108}^{2}=26.6$, p < 0. 001, η^2^ = 0. 25) (Figure 6). *Post hoc* comparison for COV_5–20_ as well as *RI* indicates that APS and HD groups showed significantly higher instability of syllable repetition than HC and PD groups. In addition, *post hoc* comparison of RA demonstrated that the PD group tended to significantly accelerate rhythm in comparison to the HC, EP, and HD groups. Although not significant, a similar trend toward acceleration of rhythm was also observed in PSP and MSA; when comparing the performance of individual speakers to the 5–95th percentile based on the HC group, pace acceleration (RA >0.45) was observed in 11 PD (50%), 4 MSA (44%), and 5 PSP (45%) patients, and only in 2 EP (8%), 1 HD (5%), and 1 HC (4%) speakers.

In the PD group, RA showed significant correlation to disease duration (*r* = −0.53, *p* = 0.01). In the pooled APS group, the NNIPPS score correlated with both RI measures of COV_5–20_ (*r* = 0.42, *p* = 0.005) and RI (*r* = 0.41, *p* = 0.006). Similarly, correlations between the NNIPPS score and COV_5–20_ (*r* = 0.59, *p* = 0.01) as well as RI (*r* = 0.76, *p* < 0.001) were observed in the HD group. No other significant correlations were detected between rhythm variables, motor severity scales, and disease duration.

## Discussion

In the current study, we present a fully automatic approach to assess rhythm in dysarthrias based upon a syllable repetition paradigm. Our algorithm was able to correctly identify syllables and remove error detections, such as excessive inspirations and non-speech sounds, with a very high accuracy of 99.6%. The newly proposed features proved capable of describing rhythm abnormalities in dysarthrias associated with basal ganglia dysfunction. According to our data, impairment of steady vocal pace performance can be observed in HD as well as all investigated APS including PSP, MSA, and EP. Significantly increased pace acceleration was observed only in the PD group. Although not significant, a tendency for pace acceleration was also observed in the PSP and MSA groups.

Our findings on rhythm abnormalities are in general agreement with previous research demonstrating impairment of vocal pace stability in PD, PSP, and HD (Skodda et al., 2010, 2012, 2014). Although impaired steadiness of syllable repetition has been reported even in the early motor stages of PD (Skodda, 2015), we observed only a non-significant trend toward RI in PD subjects. One possible explanation is that our PD patients profited from long-term dopaminergic medication, which could resulted in improvement of their speech performance. However, impairment of steady syllable repetition has been found to be unresponsive to levodopa-induced ON/OFF fluctuations (Skodda et al., 2011b). Interestingly, the greatest RI was found in MSA, EP, and HD groups, probably as a result of dominant hyperkinetic dysarthria in EP (Rusz et al., 2014a) and HD (Rusz et al., 2014b), and ataxic dysarthria in MSA (Rusz et al., 2015). Indeed, hyperkinetic and ataxic dysarthria typically induce excessive vocal fluctuations (Rusz et al., 2014b, 2015), which may greatly affect vocal pace stability.

Our PD speakers manifested accelerated rhythm. In addition, PSP and MSA patients showed a tendency for accelerated rhythm, although this finding was not statistically significant, likely due to the small sample size. Pace acceleration was not observed in EP and HD subjects. The observed RA in PD concurs with previously reported oral and gait festination (Moreau et al., 2007). However, festinating gait is not specific for PD and may also be encountered in neurodegenerative parkinsonism, such as PSP or MSA (Factor, 2008; Grabli et al., 2012), and therefore one could expect a pattern of speech acceleration in APS if there are similar pathogenic mechanisms responsible for gait and speech festination. In addition, we observed significant correlation between the extent of RA and disease duration in our PD group. Accordingly, the relationship between severity of pace acceleration and motor deficits has also been previously reported in PD (Skodda et al., 2010). These findings generally suggest a higher occurrence of oral festination in later stages of the disease.

It thus remains to be elucidated by what mechanism the basal ganglia contribute to the occurrence of oral festination. As pace acceleration was observed in PD as well as PSP and MSA, we may hypothesize that oral festination is specific for parkinsonism related to presynaptic or postsynaptic involvement of the nigrostriatal pathway. The fact that pace acceleration was not seen in the EP group may be related to a predominant involvement of the globus pallidus in EP (Selikhova et al., 2008). Another hypothesis may be that dysarthria of PD, PSP, and MSA is primarily hypokinetic, whereas both EP and HD can be characterized by the occurrence of dominant hyperkinetic dysarthria due to a predominance of dystonia in EP and chorea in HD, which may substantially influence speech manifestations, such as RA. In addition, we did not stratify our patients according to laterality dominance, while acceleration of syllable repetition appears to be more pronounced in patients with left-dominant motor manifestations (Flasskamp et al., 2012). Further studies are therefore necessary to elucidate the role of the basal ganglia and specific neural structures in oral festination.

It is noteworthy to point out that the current results are based on a simple syllable repetition paradigm and may not be compared with complex speech task, such as monolog, which has been shown to be superior to automated stimuli and more likely to elicit various speech deficits (Vogel et al., 2012; Rusz et al., 2013a). Nonetheless, both simple and highly complex speech tasks rely upon the integrity of basic motor speech programs, and a simple paradigm, such as syllable repetition, may provide a useful method to capture rhythm disorder in dysarthrias that does not require a multi-layered approach to characterizing rhythmic performance, such as during connected speech. Indeed, using a range of acoustic rhythm metrics and speech tasks, Lowit (2014) did not detect any differences between healthy and disordered speakers, although disordered speakers were perceptually identified to manifest rhythmic deviations. This finding clearly suggests that it is not sufficient to only capture duration-based characteristics without considering how these are related to fundamental frequency and intensity production in creating the rhythmic patterns of speech (Lowit, 2014).

In the present study, the algorithm developed for the automated identification of syllables reached a very high classification accuracy of 99.6%. Moreover, rhythm features based on the automatic algorithm exhibited strong correlation with the results obtained using manual labels suggesting the reliability of the proposed algorithm in clinical practice, as it is more important to achieve a correct estimation of rhythm performance than to obtain the precise position of syllable nuclei. The accuracy of our algorithm cannot be compared to previous methods as this study provides the first attempt toward automatic evaluation of rhythm in dysarthria based on a syllable repetition paradigm. Although a number of previous studies strived to provide methods for automatic identification of syllable nuclei (Mermelstein, 1975; Xie and Niyogi, 2006; Wang and Narayanan, 2007; De Jong and Wempe, 2009), these methods were designed for connected speech, particularly for the estimation of speech rate, and their reliability was tested using recordings of healthy speakers. Nonetheless, one might assume that identification of isolated syllables from syllable repetition paradigm is a rather simple task when compared to detection of syllables nuclei from continuous speech. However, when considering syllable repetition paradigm, there is still a need for precise syllable nuclei identification as just one missed or false-detected syllable may lead to substantial distortion of resulted rhythm metrics.

Although the inclusion criterion for participants was to be able to sustain phonation for at least 6 s to ensure that results were not influenced by severe respiratory problems, the most challenging part of the algorithm design was to avoid the erroneous identification of excessive inspirations and non-speech sounds counted as syllables. Difficulties with audible inspirations occurred particularly in HD patients, which are in agreement with the severe respiratory problems typically observed in HD (Rusz et al., 2013b). Non-speech sounds were mainly present in APS and HD patients, likely as a result of greater disease and dysarthria severity. Nevertheless, excessive inspiration still may prolong the interval between two subsequent syllables and thus contribute to greater pace instability, even if it is correctly detected and not included in further analysis. Indeed, we have found correlation between motor severity scores and RI for the APS as well as HD groups, suggesting that the precision of syllable repetition steadiness is substantially influenced by overall disease severity.

We further strived to elaborate and provide more robust variant of features previously designed to evaluate the aspects of speech RI and RA (Skodda et al., 2010). In particular, PA can be dependent on the length of the reference interval obtained from the first to fourth syllable. As an example, when comparing two speakers with slower and higher PR at the beginning (reference interval), the resulting PA value will be always lower for the speaker with a slower PR even if both speakers maintain the same acceleration velocity. Moreover, even the random occurrence of inspiration or other longer pauses into subsequent syllable groups (5–12th or 13–20th) may cause substantial random influence on PA. The description of the acceleration using the gradient of the regression line (RA) benefits from the entire speech sample and therefore provides robust estimation independent of the speech rate and rhythm fluctuations. Conversely, COV_5–20_ represents the SD of the 5–20th syllable weighted by the reference interval. Ideally paced but accelerated rhythm will show a higher SD, similar to constant but unstable rhythm, and therefore it cannot be ensured that a higher COV_5–20_ is related to greater RI rather than RA. Expression of RI through absolute deviations of the syllable gap lengths from the regression line (RI) assures a measurement independent from speech rate and acceleration.

The current study has certain limitations. Our algorithm was tested only using /pa/ syllable repetition. As the algorithm was robustly designed to detect spectrally distinctive nuclei of repetitive syllables, we believe that it is applicable to other syllables as well; however, we cannot exclude that certain optimization will be necessary. One optimization of the current algorithm for future applications may consist of preprocessing of speech signal using low-cut filter for removing non-deterministic low frequency noise from recorded signals. Subsequently, algorithm accuracy was tested using all available data and we did not perform the validation of the current algorithm using separated dataset. Nevertheless, algorithm was designed based on the model of rhythm task without using supervised training of classifier and tuning of algorithm threshold parameters.

The present study provides a novel extension of available technologies for the automatic evaluation of various dysarthric features. In particular, objective investigation of certain speech patterns can raise suspicion regarding the etiology of disease and may be diagnostically helpful in a number of neurological disorders. Previous research has shown that PD patients manifest a tendency for pace acceleration, which was not present in speakers with cerebellar ataxia (Schmitz-Hubsch et al., 2012). Currently, we have shown that a tendency for RA is specific for neurodegenerative parkinsonism and can be found in PD, PSP, and MSA, while vocal pace fluctuations occur mainly as a consequence of hyperkinetic and ataxic dysarthria. Our findings underline the crucial role of the basal ganglia in the performance and the maintenance of automatic speech motor sequences.

## Conflict of Interest Statement

The authors declare that the research was conducted in the absence of any commercial or financial relationships that could be construed as a potential conflict of interest.
